# The Efficacy of Allergen Immunotherapy with Cat Dander in Reducing Symptoms in Clinical Practice

**DOI:** 10.1155/2013/324207

**Published:** 2013-07-31

**Authors:** Aerik A. Williams, John R. Cohn, Shirley M. Fung, Patricia Padams

**Affiliations:** Thomas Jefferson University, USA

## Abstract

*Background*. Allergy to cat dander is a common form of allergic disease. Allergen immunotherapy has been demonstrated to be effective in decreasing allergic symptoms. *Objectives*. To examine outcomes in allergic asthmatic patients on cat immunotherapy (CIT) compared to allergic asthmatics on traditional immunotherapy (IT) without cat sensitivity. *Methods*. A retrospective review identified allergic asthmatics on CIT for at least three years. An equal number of allergic asthmatics on IT were identified for comparison. Outcomes investigated include measurements of risk of asthma exacerbation. *Results*. Thirty-five patients were identified in each group. There were no differences in the CIT group versus the comparison group regarding total number of prednisone tapers (18 tapers versus 14 tapers, resp.), number of patients requiring prednisone tapers (10 patients versus 10 patients, resp.), total number of acute visits (29 visits versus 38 visits, resp.), and number of patients requiring acute visits (15 patients versus 21 patients, resp.). When stratified by concomitant ICS use, patients on CIT were less likely to require an acute visit (46% versus 78%, resp.). *Conclusions*. Allergic asthmatics with cat sensitivity on CIT with close dander exposure have similar risk of asthma exacerbation compared to allergic asthmatics without cat sensitivity on immunotherapy.

## 1. Introduction

Allergen injection immunotherapy with cat dander extract (CIT) is an effective treatment for allergic bronchial asthma in acute challenge models of cat allergy, but there is limited data concerning the efficacy of CIT in the clinical setting [1, 2].

Indications for allergen immunotherapy in a patient with allergic asthma include increased symptoms after exposure to the allergen demonstrated evidence of clinically relevant specific IgE antibodies and one of the following: poor response to pharmacotherapy or allergen avoidance, desire to avoid long-term pharmacotherapy, or coexisting allergic rhinitis [3]. Allergy to pets presents unique management issues. The theoretical opportunity exists for the cat allergic patient to remove the source of allergen from the home environment. However patients and their families are emotionally attached to pets and often resist recommendations to remove them.

A pet cat or cats in the home are associated with high levels of allergen exposure and increases in nonspecific airway hyperreactivity [4, 5]. While persistent symptoms even with a change in environment have been demonstrated, there is little direct evidence to answer the common patient question: “Is there a way I can safely keep the cat?” [6].

The purpose of the following study is to compare clinical outcomes of allergic asthmatic patients with cat sensitivity and close dander exposure on CIT to patients with allergic asthma on allergen immunotherapy (IT), however, without cat sensitivity.

## 2. Materials and Methods

Records reviewed were those of adult allergic asthmatic patients (18 years or older) undergoing subcutaneous immunotherapy, based on skin testing and history, for at least three consecutive years. 

This study was a retrospective chart review conducted at a single urban, university hospital-based allergy, and immunology practice. It was approved by the Thomas Jefferson University Institutional Review Board. We evaluated measures of clinical outcomes in allergic asthmatic patients on continuous immunotherapy during the period of January 2005 through December 2008. Clinical outcomes were assessed during the second and third years of immunotherapy. 

The study group consisted of cat allergic asthmaticpatients as determined by skin prick or intradermal skin test to Fel d1 (Greer's Standardized Cat Hair, dose 10,000 bau/mL and 100 bau/mL respectively) with perennial, usually home, exposure to cat dander. Skin prick and intradermal testing results were determined by one of two allergists. Asthma was defined as a clinical diagnosis made by one of two allergists documented in the patient's chart. CIT was administered with standardized cat hair extract according to the manufacturer's label (Greer Laboratories).

An equal number of allergic asthmatic patients with no cat sensitivity on subcutaneous IT during the same time period were randomly selected to serve as controls. Allergen extracts for noncat allergens were selected according to conventional practice, based on skin test results, patient history, and prevalent regional allergens. All patients avoided antihistamines at least five days prior to skin testing. 

FEV1, a marker of asthma impairment, was recorded in both groups as well as age, gender, inhaled corticosteroids (ICS) use, duration of immunotherapy, and previous diagnosis of allergic rhinitis. 

Outcome variables investigated are markers of risk for an asthma exacerbation and include the following: prednisone tapers, acute outpatient visits for asthma-related symptoms, and hospitalizations for asthma-related symptoms during the second and third years of immunotherapy. In a subgroup analysis, patients on concomitant ICS were compared using the same outcome variables.

*Statistical Analysis.* Statistical analyses were carried out using STATA version 10.0 statistical software (Stata Corp, College Station, TX, USA). Comparisons between groups were made using the *t*-test and *χ* ^2^ test for continuous and categorical variables, respectively. 

## 3. Results and Discussion

### 3.1. Results

A total of 70 patients were included in these analyses. Thirty-five patients with allergic asthma, perennial cat exposure, positive skin prick or intradermal test to standardized cat extract, and undergoing subcutaneous CIT ± other subcutaneous immunotherapy were identified. An equal number of patients with allergic asthma undergoing subcutaneous IT, but where significant cat allergy and exposure were not found to be present, were identified as well and served as the comparison group. As will be discussed in more detail, and as the demographics confirm, the reasoning behind this choice was to identify an asthma patient subset of similar baseline impairment but without cat allergy playing an important role. 

There was no difference in gender or age between the two groups (Table 1). All patients had a minimum of three years of immunotherapy and there was no difference in average duration of immunotherapy between the CIT group (5.2 years) and the IT group (5.5 years) (*P* = 0.36). Percentage of FEV1 (CIT = 84.4%, IT = 86.6%; *P* = 0.66) and ICS use prior to initiation of immunotherapy (26 patients in CIT group versus 23 patients in IT group; *P* = 0.43) was not significantly different. The prevalence of allergic rhinitis was similar among patients in both groups (CIT = 97%, IT = 100%; *P* = 0.31). 

Patients in the CIT group were significantly less likely to be receiving subcutaneous pollen (46% versus 71%; *P* = 0.02), mold (9% versus 43%; *P* = 0.001), and dust/mite (57% versus 94%; *P* = 0.001) immunotherapy compared to patients in the IT group (Table 2). Presumably, this reflected genetic and/or exposure differences in propensity to sensitization by these various allergen groups. There was no significant difference in the number of patients requiring prednisone tapers (*P* = 1.0), total number of prednisone tapers (*P* = 0.58), number of patients requiring an acute office visit for asthma symptoms (*P* = 0.15), total number of acute office visits for asthma symptoms (*P* = 0.38), and number of hospitalizations between the two groups (*P* = 0.31). 

In a subgroup analysis considering only patients on concomitant ICS, there were twenty-six patients undergoing CIT ± other subcutaneous immunotherapy and twenty-three patients undergoing IT (Table 3). There was no difference in gender, age, baseline percentage FEV1, duration of immunotherapy, or history of allergic rhinitis. Patients in the CIT group were less likely to be receiving subcutaneous pollen (42% versus 83%; *P* = 0.004), mold (12% versus 57%; *P* = 0.001), and dust/mite (50% versus 96%; *P* = 0.001) immunotherapy compared to patients in the IT group. Unfortunately, our data does not allow us to further elucidate why; for example, patients symptomatic with cat induced allergic asthma were less likely to have pollen allergy. This may be another manifestation of the conflicting data on the effect of animals in the home on the development of symptomatic atopy. Patients on CIT and ICS were less likely to require an acute office visit for asthma symptoms as compared to patients on IT and ICS (46% versus 78%; *P* = 0.02) (Table 4).

### 3.2. Discussion

Allergic asthmatic patients with cat allergy and perennial exposure to cat dander showed benefit from CIT. Asthmatic CIT patients on ICS were less likely to require an acute office visit for asthma-related symptoms compared to allergic asthmatic patients on IT and concomitant ICS although they had similar baseline characteristics. An improvement in asthma symptomatology—specifically improvement in bronchial sensitivity and hyperresponsiveness to histamine—in patients on both allergen specific immunotherapy and concomitant inhaled corticosteroids has been reported in a pediatric population [7]. To our knowledge, this is the first report comparing clinical outcomes in adult patients on particular allergen immunotherapy and concomitant ICS.

There has been a significant amount of data detailing the immune response to CIT; however, evidence supporting clinical benefit is sparse [8–10]. This study is important because it demonstrates some improvement in clinical parameters in patients on CIT and concomitant ICS compared to patients on IT and concomitant ICS. While this seems inexplicable, it may reflect another aspect of the proposed protective effect of animals in the home on atopic disease. That is well beyond the scope of this report but raises the intriguing question as to whether cat induced asthma was somehow protective as it relates to other allergic disease.

This hypothesis would also be supported by the fact that cat allergic patients were less likely to be receiving other extracts. Alternatively, this may reflect antigen selection based on the most likely antigens to be causing a particular patient's symptoms, but it is of interest that the two groups, so similar in many ways, differed significantly in the other antigens that were thought clinically significant.

Clinicians are commonly confronted with cat owners who are adamantly opposed to eliminating the cat from their home environment. The persistence of cat allergen complicates management of the cat-sensitive asthmatic patient. The allergen may be present for several years even after the cat is displaced from the environment [11, 12]. Preventative measures including cat washing, keeping cats outside of bedrooms and the utilization of air filtration may be of little-to-no benefit [11, 13–15]. Our data suggest that cat owners on CIT do equally well as other asthmatic patients also undergoing immunotherapy, but without cat allergy and potent cat antigens inside their homes. This suggests that patients may indeed safely keep their cats, at least if they do so in association with a course of allergen immunotherapy to cat antigen.

A key question is whether keeping the cat will result in long-term, irreversible harm, as seen in other exposure models [16, 17]. Pulmonary epithelial damage in the asthmatic patient allows allergen and other toxins to precipitate in the airway thus augmenting inflammation, leading to airway remodeling [18]. In mice, chronic exposure to house dust mite has demonstrated a disturbance of the epithelial-mesenchymal trophic unit and increased production of deleterious mesenchymal proteins in the large airway [19]. There is also evidence of increased levels of inflammatory proteins and upregulation of remodeling genes following allergen exposure, with persistence of remodeling proteins for at least seven days [20]. A next investigative step would be to determine if airway remodeling, a pathophysiologic process of increasing importance, is inhibited by allergen IT in patients on appropriate specific IT. 

There are limitations to note in this study. The authors would be remiss if not to report that this study was underpowered (69%) when considering the significant finding of asthmatic CIT patients on ICS being more likely to require an office visit. In addition, patients in the CIT group were less likely to be on pollen, dust/mite, and mold immunotherapy compared to the comparison group. While we have suggested intrinsic differences in the groups, one could suggest that these groups are therefore not similar in other ways, unrelated to cat exposure and sensitization. In the authors' opinion, it is not the number of antigens selected for immunotherapy that is indicative of the severity of disease, but rather the decision to proceed with immunotherapy itself. 

Markers of bronchial responsiveness including PC 20 histamine challenges and allergen challenges are frequently used when evaluating the effect of immunotherapy on asthma control. This data was not available for our study. However, our outcome variables were reflective of those commonly used to assess clinical response. Another limitation to note concerns selection of participants into the study. Cat allergic patients may be more motivated to undergo any treatment that is intended to reduce the likelihood they will need to eliminate a beloved pet.

Ideally, a randomized, double blind placebo control study would better elucidate the effect of CIT on cat exposed sensitized patients. Such a study is unlikely to be undertaken. Regarding duration of treatment needed, it would require several years with half the subjects getting placebo. In light of the availability of subcutaneous immunotherapy and the data on efficacy in general and CIT specifically, patient recruitment would be problematic and the expense of such a study might be difficult to justify. 

For this reason we selected a comparison group of similar symptomatic subjects and similar asthma impairment, but where cat allergy did not play an important role, and to whom cat extract was not administered. All subjects were followed closely. While this was a retrospective review, they were all seen and evaluated regularly, and the parameters selected for evaluation were of the type usually well documented in patient records.

## 4. Conclusion

We found asthmatic patients with allergy to cat and regular cat exposure do as well or better on CIT than similarly ill asthma patients receiving allergen immunotherapy but where cat allergy is not considered clinically important. While chronic allergen exposure has been shown to have deleterious effect, including enhanced airway remodeling, cat allergic patients treated with specific CIT may be protected from adverse outcome. 

## Figures and Tables

**Table 1:** Patient characteristics.

	Cat immunotherapy *n* = 35	Other immunotherapy *n* = 35	*P* value
Gender, *n* (%)			
Male	14 (40%)	17 (49%)	
Female	21 (60%)	18 (51%)	0.47
Age (years)			
Mean (SD)	49.3 (14.7)	55 (13.6)	0.11
Range	29–86	28–89	
Baseline FEV1 (%)			
Mean (SD)	84.4 (21.8)	86.6 (17.9)	0.66
Range	38–117	43–127	
History of allergic rhinitis	34 (97%)	35 (100%)	0.31
Mean duration immunotherapy (years) (SD)	5.2 (1.9)	5.5 (2.5)	0.36
Inhaled corticosteroids *n*, (%)	26 (74%)	23 (66%)	0.43
Treatment antigens *n*, (%)			
Dog	10 (29%)	9 (26%)	0.79
Pollen	16 (46%)	25 (71%)	0.02
Mold	3 (9%)	15 (43%)	0.001
Dust/mite	20 (57%)	33 (94%)	0.001

Allergic asthmatic patients on cat immunotherapy or traditional immunotherapy.

Comparisons between groups were made using the *t* test and *χ* ^2^ test where appropriate.

**Table 2:** Clinical outcomes in patients on CIT and IT.

	Cat immunotherapy *n* = 35	Other immunotherapy *n* = 35	*P* value
Patients requiring prednisone taper, *n* (%)	10 (29%)	10 (29%)	1.00
Prednisone tapers, *n*	18	14	0.58
Patients requiring acute office visit, *n* (%)	15 (43%)	21 (60%)	0.15
Acute office visits, *n*	29	38	0.38
Hospitalizations, *n* (%)	1 (3%)	0 (0%)	0.31

Allergic asthmatic patients on cat immunotherapy or traditional immunotherapy.

Comparisons between groups were made using the *t* test and *χ* ^2^ test where appropriate.

**Table 3:** Characteristics of patients on inhaled corticosteroids.

	Cat immunotherapy *n* = 26	Traditional immunotherapy *n* = 23	*P* value
Gender, *n* (%)			
Male	10 (38%)	10 (43%)	0.72
Female	16 (62%)	13 (57%)	
Age (years)			
Mean (SD)	49 (13.7)	56 (12.6)	0.05
Range	29–75	36–80	
Baseline FEV1 (%)			
Mean (SD)	80.8 (21.5)	85.0 (20.4)	0.49
Range	38–112	43–127	
History of allergic rhinitis	25 (96%)	25 (100%)	0.34
Mean duration immunotherapy (years) (SD)	5.2 (2.0)	5.7 (2.3)	0.62
Treatment antigens, *n* (%)			
Dog	7 (27%)	6 (26%)	0.94
Pollen	11 (42%)	19 (83%)	0.004
Mold	3 (12%)	13 (57%)	0.001
Dust/mite	13 (50%)	22 (96%)	0.001

Allergic asthmatic patients on inhaled corticosteroids and immunotherapy

Comparisons between groups were made using the *t* test and *χ* ^2^ test where appropriate.

**Table 4:** Clinical Outcomes in Patients on Concomitant ICS.

	Cat immunotherapy *n* = 26	Traditional Immunotherapy *n* = 23	*P* value
Patients requiring prednisone taper, *n* (%)	10 (38.4%)	10 (43%)	0.72
Prednisone tapers	18	14	0.76
Patients requiring acute office visit, *n* (%)	12 (46%)	18 (78%)	0.02
Acute office visits	26 visits	34 visits	0.21
Hospitalizations, *n* (%)	1 (3.8%)	0 (0%)	0.34

Allergic asthmatic patients on inhaled corticosteroids and immunotherapy.

Comparisons between groups were made using the *t* test and *χ* ^2^ where appropriate.
